# Supplementary figures and images for: Mediating artificial intelligence developments through negative and positive incentives
Source: PLoS One. 2021 Jan 26;16(1):e0244592. doi: 10.1371/journal.pone.0244592 (PMC7837463; doi:10.1371/journal.pone.0244592)

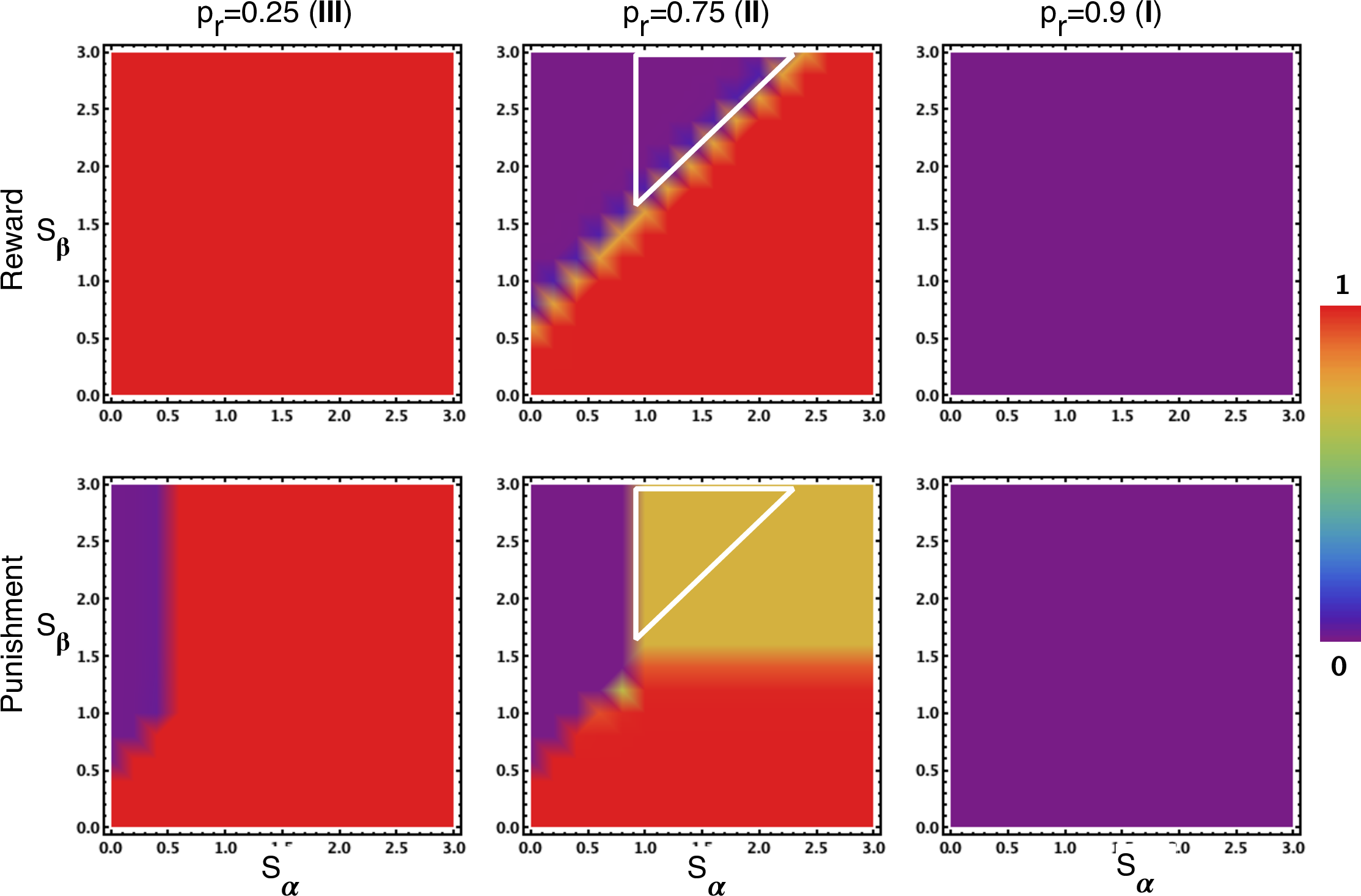

Supplement: S1 Fig — Other parameters are the same as in Fig 5 in the main text. The observations in that figure is also robust for larger intensities of selection. (TIF) [file pone.0244592.s001.tif]

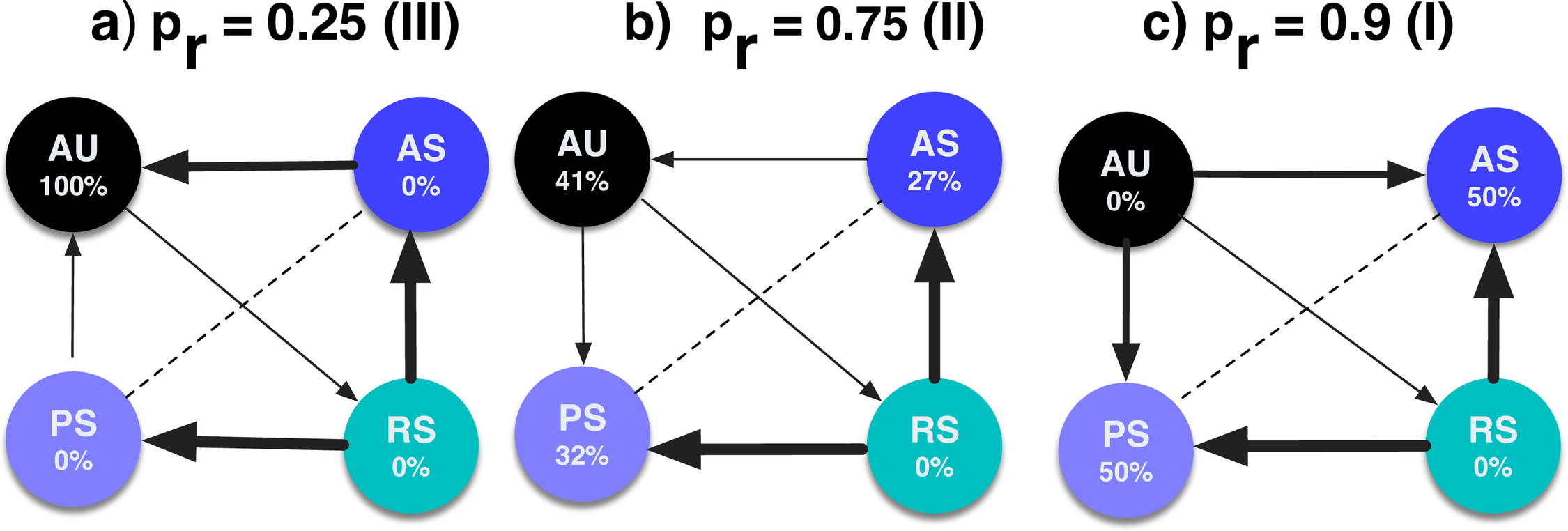

Supplement: S2 Fig — Only stronger transitions are shown for clarity. Dashed lines denote neutral transitions. In addition, note that PS is equivalent to AS when interacting with PS, i.e. there is always a stronger transition from RS to PS than vice versa. Parameters as in Fig 2. (TIF) [file pone.0244592.s002.tif]

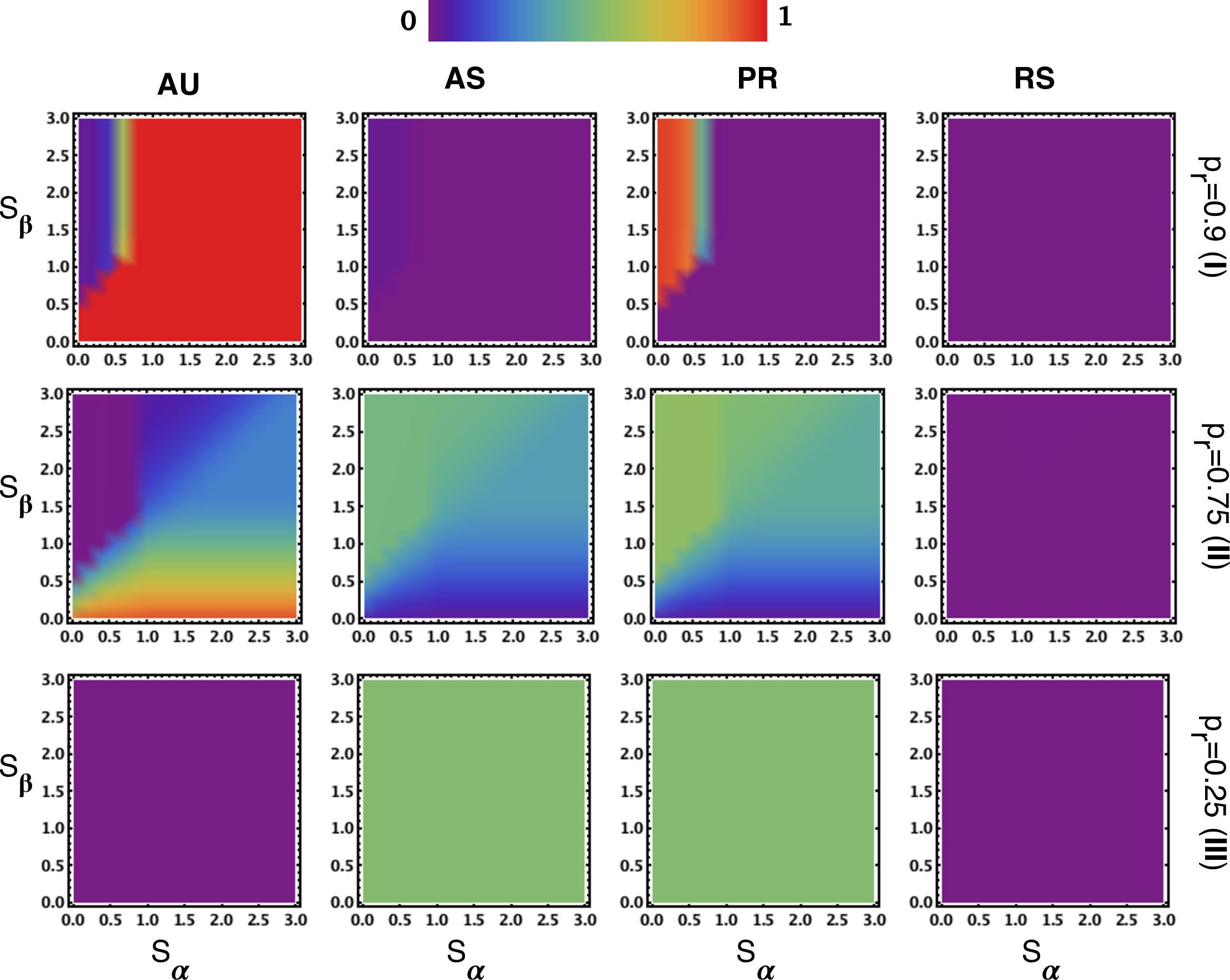

Supplement: S3 Fig — The outcomes in all regions are similar to the case of punishment (without reward) in Fig 5. The reason is that there is always a stronger transition from RS to PS than vice versa. Parameters as in Fig 5. (TIF) [file pone.0244592.s003.tif]

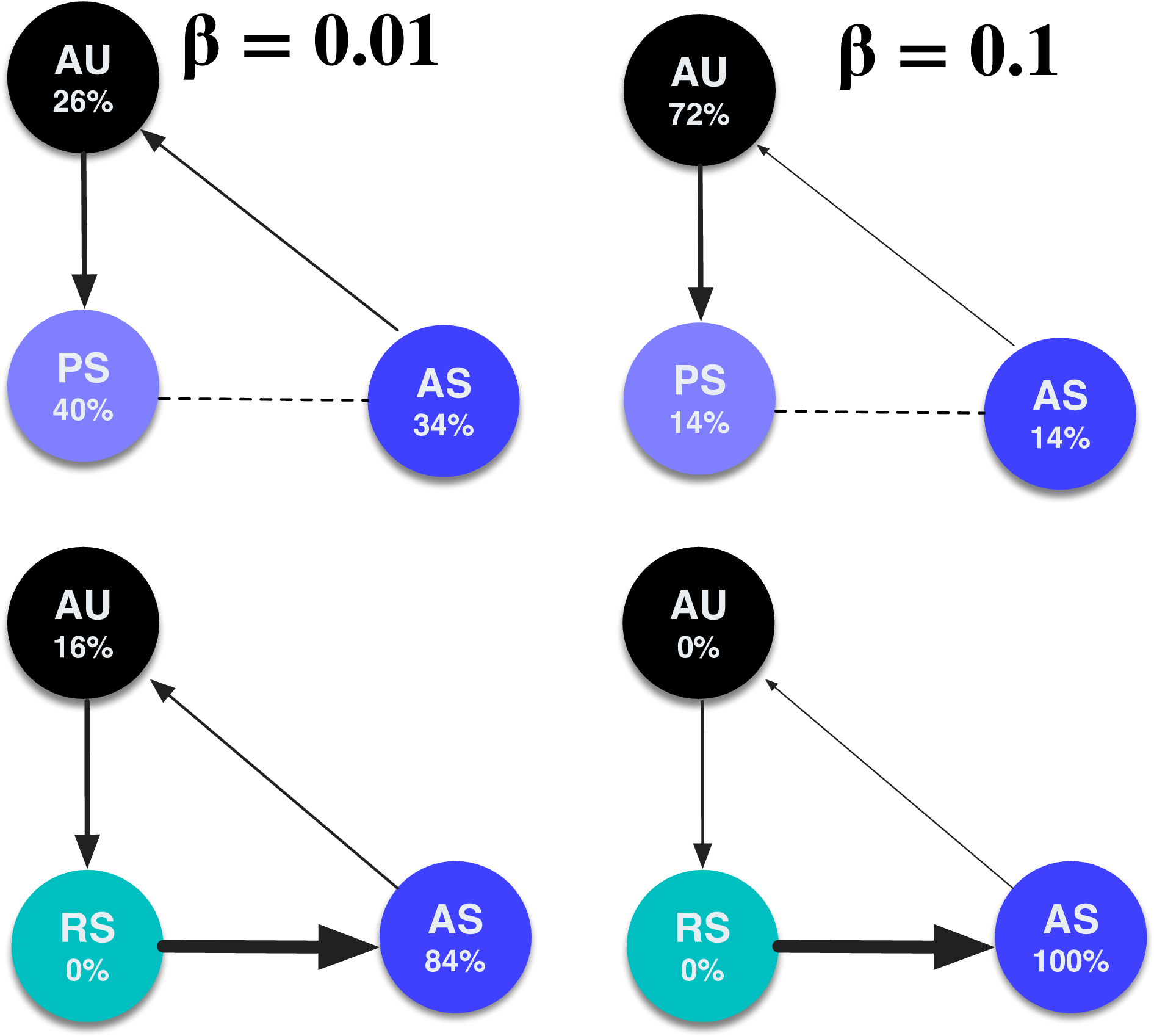

Supplement: S4 Fig — The parameters of incentives fall in the white triangles in Fig 5 and S1 Fig: sα = 1.5, sβ = 3. We observe that the frequency of AU is lower in case of reward than that of punishment. Other parameters as in Fig 2. (TIF) [file pone.0244592.s004.tif]
