# Supplementary material for: Mediating artificial intelligence developments through negative and positive incentives
Source: PLoS One. 2021 Jan 26;16(1):e0244592. doi: 10.1371/journal.pone.0244592 (PMC7837463; doi:10.1371/journal.pone.0244592)
Supplement: Attachment — Submitted filename: Response_to_Reviewers.pdf [file pone.0244592.s006.pdf]

Detailed Reponses to Reviewers

We thank all the reviewers for spending time carefully reading our manuscript and for the constructive comments and suggestions. This allowed us to produce a significantly improved version of the manuscript. You can find below a point-by-point responses to all comments and suggestions made, and how they are addressed in the revised manuscript. We also attached a revised version of the manuscript where all changes are highlighted (typed in red). Thank you, once again.

Reviewer #1:

In the manuscript, the authors revealed the effects of rewards and punishments on AI developments (or other technology development races). They analytically and numerically solved the dynamics among some strategies (AS, AU, PS, and RS) by the means of Evolutionary Game Theory (EGT) and found that both rewards and punishments are effective for the developments.

I think the most contribution of this manuscript and Ref. 13 is that the authors invented the payoff matrix for the AI developments based on game theory. The payoff matrix is different from the one in social dilemma games, which EGT has frequently been applied. Although the original matrix was invented in Ref. 13 by the same authors, the authors included new strategies related to rewards and punishments in this manuscript. Thus, they successfully extended the study of this AI development games.

I recommend the publication of this manuscript in PLoS ONE after the following technical points are improved.

R: We thank the reviewer for the positive assessment of our manuscript and for his/her willingness to accept it for publication. We are also grateful for all the generous suggestions provided. Below please find a response to all her/his comments and details of the modifications introduced.

There are two points I find the authors can improve.
1. Organization of the manuscript. I think the authors can rearrange the structure of the manuscript to be better read.

Examples are below.
- The sixth and seventh paragraphs (lines 46-71) in the introduction can be combined with the fifth paragraph (lines 134-142) in the Materials and methods.

R: Thank you for pointing this out. We have now re-organized the two paragraphs in the introduction and moved the more technical parts regarding the names of the parameters regions, to the Methods section.

- When I read the model, especially Eq. 2., I forget what B and W are. Those variables are defined in the sixth paragraph (lines 46-57) in the Introduction, which is far from the model section. Perhaps, the Related Work section can be moved to the earlier part of the Introduction?

R: We agree. We have now rearranged the Related Work section, moving part of it to early part of the introduction and the other part to Discussion section. We also made sure to recall the meaning of these variables throughout the text. Thank you raising our attention to this issue.

In short, if the authors could restructure the Introduction, Related Work, and Model, it would be beneficial to readers for better understanding.

2. Numerical calculations. It is better to give the detail procedures of the numerical calculation. I roughly understood how the analytical solutions were derived. However, I don't know how the numerical one is obtained. For example, in Figs. 2 and 4, the white lines are analytically obtained by calculating the fixation probabilities of strategies. A fixation probability represents the PROBABILITY that a single mutant replaces the existing population. However, the color maps in Figs. 2 and 4 obtained by the numerical calculations represents the FREQUENCY of the strategies. I don't know how the frequency is calculated. Did you numerically get the frequency by iterating Eq. 4 until the stationary distributions are obtained? If the authors give the detail procedure of the numerical calculations as a subsection, it would be great.

R: Thank you for your comment. We have revised the text to clarify our methodology and how we analyze this stochastic process.

The lines in Figs. 2 and 4 are obtained without having to calculate fixation probabilities. It is based on Eq. (6) in the Methods section, which allows ones to determine analytical conditions when a strategy is risk-dominant against another one in the limit of large population size. We have now explicitly added that information in Supporting Information (before Equation 16). Thanks for pointing this out.

Numerical calculation is done following closely the steps written in the revised version of our Methods section and pages 5 and 6. Your comment clearly suggests that our explanation does not have enough detail. As a result, we have improved this part in the revised version.

Here, individuals revise their strategies through social learning: each time-step an individual is given the chance of imitating another individual with a probability that increases with their fitness difference. This is the so-called pairwise comparison rule introduced in page 5. We further assume that with a probability μ individuals switch to a randomly chosen strategy, freely exploring the space of possible behaviors.

In the absence of exploration, the end states of evolution are inevitably monomorphic (i.e., the entire populations adopts a single strategy), as a result of the stochastic nature of the evolutionary dynamics and update rule. By introducing a small probability of exploration, the population will either end up wiping out the mutant or witness the fixation of the intruder. In other words, in this limit, the mutant will fixate or will become extinct long before the occurrence of another mutation. This is often referred in the literature as the small-mutation or exploration limit, which has been shown to have a range of applicability which goes well beyond the strict limit of very small mutation (or exploration) rates.

Importantly, it allows one to describe the evolutionary dynamics of our population in terms of a reduced Markov Chain with a size given by the number of strategies: Each state represents a possible monomorphic state associated with a given strategy, and the transitions between states are defined by the fixation probabilities of a single mutant of one strategy in a population of individuals who adopt another strategy. The resulting stationary distribution of this Markov chain characterizes the average time the population spends in each of these states, and can be computed analytically as an eigenvector problem (see Methods).

In particular, as described in the Methods, to compute this stationary distribution, we need to use Equation 4 to calculate the fixation probabilities of a single mutant adopting a strategy A in resident population of B's, for all pairs of strategies A and B. From these probabilities, we

then form the transition matrix M (last paragraph in Methods) of this reduced Markov Chain and calculate a stationary distribution, which determines the (long-term) frequency of strategies in the population, as reported in Fig 3. Figs 2 and 4 report the frequency of AU only.

We have extended the description of procedure to further clarify the abovementioned details (please see Methods section).

The followings are minor points.
- Could you add an intuitive explanation that why the risk-dominance thresholds are obtained only by two variables, p_r and s? I mathematically understand, if B/W is large enough, some variables can be ignored, but I don't know how it can be interpreted?

R: The intuition is that, if B/W is sufficiently large, the average payoff obtained from winning the race (i.e. gaining B) is significantly larger than the intermediate benefit a player can obtain from each round of the game (at most b), making the latter irrelevant. Thus, in this scenario (early DSAI), the only way to improve a player's average payoff (i.e. individual fitness) is to increase the player's speed of gaining B. On the other hand, AU's payoff is scaled by a factor (1-p_r). A good way of better understanding this is to look at equation 11: since \pi_11 and \pi_22 become irrelevant, they can be removed. Following your question, we have added a paragraph in SI to discuss this (below Equation 16).

- Please add the clear definition of B. Lines 47 and 53 give the relation to that point but is not clearly defined. Does B imply the net benefit of the AI achievement?

R: The definition of B is given on lines 36-37. We have also recalled this definition in other parts of the text, to make the text easier to follow. B stands for the prize of winning the whole (domain) AI development race, which is assumed to be large. It's the benefit/prize a team/company can gain when becoming the first to have successfully developed and deployed an AI technology (such as image/voice/emotion recognition, natural language processing, etc)

- Line 315. "dynamics ." The space should be removed.

R: Corrected, thanks.

Once again, we would like to thank the reviewer for all his/her suggestions and for the positive assessment of our contribution. Thank you!

Reviewer #2:

This is a very well written paper with a clear and concise model. My only suggestion would be to potentially enlarge the discussion. I believe the incentives discussed probably also apply to fields other than AI. Vaccine development or biotech in general, comes to mind.

I strongly recommend this paper is published.

We thank the reviewer for the appreciation of our work and for sharing our enthusiasm with our model. Following your suggestion, we have extended our discussion to include incentives in other fields. Namely, indeed there have been research on efficient use of incentives for improving vaccination behaviour, which have been now mentioned and cited in our revised manuscript.. Moreover, similar to patent racing, innovation dynamics in biotech is also highly relevant to our models' scope. This is also mentioned in in our newly revised Discussion section.

Once again, thank you so much for your recommending the acceptance of our paper.

Reviewer #3:

This paper examines an evolutionary game theoretic model of a technological innovation race to study the effects of peer rewarding/punishment mechanisms. The technical part (mathematical modeling and analysis) seems sound and well presented, which should be worth publication in PLOS ONE.

R: We thank the reviewer for the appreciation of our work and for willing to accept it for publication.

Having said that, I think there are a few major issues in framing and presentation, which make the proposed model rather unjustified as a model of AI development. Details are explained below. The authors are strongly recommended to make a major revision in framing, presentation and justification of the model and its assumptions.

1. It was not clearly justified why the AI development is a particularly good interpretation of this model.

The model is a generic evolutionary game theoretic (EGT) model that represents competition among multiple players trying to reach a goal faster than the competitors while rewarding/penalizing the competitors according to their strategies. While this model setting itself is reasonably constructed and of some interest to the EGT community, it does not appear to describe the AI development scenario in particular.

R: We agree that the model proposed is definitely not the final product that captures all relevant factors in an AI development race. Instead, we propose a conceptual model, which seeks for a balance between the complexities of AI governance, and the abstraction power that a simple model of an innovation race may offer. In particular, we tried to keep the model as tractable as possible to (successfully, we hope) grasp the implications of both rewards and sanctions within a simple baseline model [see Ref. 13]. Such simplicity can be seen as both an advantage and a limitation. Particularly, it allowed us to identify the impact of incentives (peer reward & punishment) on a social dilemma pertaining to an idealized AI race, despite the specificities of each particular AI product and application, and different views on the problem. We tried to focus our discussion on the relevance of these incentives and not so much on the possible variants that could, we agree, be added to the baseline model. Nonetheless, as detailed below, we revised our manuscript to highlight some of the limitations of our baseline abstraction, in connection to the AI development problem. We hope you agree with our modifications and the listed potential extensions/limitations of the model.

Actual AI technology development companies do not engage in a series of discrete pairwise game play events with their competitors (especially given that their development stages are asynchronous due to different development speeds), and they certainly would not have any incentives to impose the proposed peer rewarding/punishment (especially rewarding) on their opponents in the real industry ecosystem. These mismatches with reality make the model questionable as a model of AI development. There may be a better analogy for interpretation of this model (e.g., rapid vaccine development in which multiple competitors are also cooperating, to some extent, to achieve a global public health goal, for example).

R: Excellent points, thank you! Following your comments, we have introduced the following changes.

1) We have added a paragraph in the Discussion to discuss the issue of asynchronous speeds, especially when players' heterogeneity (e.g. in wealth, size) is taken into account, which we aim to study in future work.

2) We also discuss the possibility of studying a continuous choice model generalizing the present binary-choice AI race model.
3) We have provided some examples of possible peer reward/punishment actions in AI/technology development, in the last paragraph of Introduction section, explicitly referring the slowing down of unsafe participants by reporting misconduct to authorities and media, or by refusal to share and collaborate with companies not following the same deontological principles. Similarly, rewards can correspond to support, exchange of knowledge, staff, etc. of safety conscious participants. Note that reasons for intervening with the development speed of competitors may also be nefarious, e.g., cyber-attacks, in order to get a speed advantage, something that is also highlighted in our revised text.
4) Finally, we also tried to highlight that our evolutionary approach, examining the viability of peer incentivizing strategies, also helps explain when such behaviours would be selected by natural selection and thus might actually be seen (often) in the real-world. One of our contributions of relevance to this area of research dedicated to the evolutionary origins institutional and peer-based (costly) incentives is that, when both peer reward and punishing options are available in a self-organized scenario, peer-punishment is more likely to prevail than peer-rewarding when individuals face an innovation race, predicting that punishment is more likely to be observed than rewards.

2. The assumptions made about the speed of development do not seem to adequately capture the reality.The key parameter of interest in this study is the speed of technology development, on which several assumptions were made: (1) The faster the speed is, the more unsafe the developed technology will be.

We thank the reviewer for pointing out this issue. There is indeed an underlying assumption here that needs clarification: We assume that all actors/participants involved in the race have equal resources/means at the start. Based on this assumption, they can only gain an advantage by increasing their speed (parameter s in the model) through unsafe actions (see also Ref [13]).

This is, of course, a simplification. While this is a natural assumption to take when agents are homogeneous in terms of resources and wealth, it may not remain true once agents become heterogeneous. For example, some teams may revise their choices or act as role models more often than others [see, e.g., Santos, Pacheco, & Lenaerts, PNAS 2006; Santos et al. Nature 2008), or react to uncertainty in different ways, leading to polarized behaviors [see, e.g., Domingos-Fernandez et al. iScience 2020 or Ross & Portugali, Royal Soc. Open Sci. 2018).

We further agree that heterogeneity may emerge from other sources. One may consider that we may have stronger teams (with more resources) than others, ensuring a greater speed than those without such advantage, even without cutting ethical/safety corners. The role of such wealth inequality can be cumbersome to study [see, e.g., (Tavoni, et al., PNAS 2011; Vasconcelos, et al. PNAS 2014)], yet would be an interesting challenge to address in the future. Such stronger teams might be able to comply regardless of the competition from weaker teams and still win the race, e.g., modifying the dynamical scenarios portrayed in Figures 1 and 3.

As the goal of this contribution is to examine the root role of positive and negative incentives, these issues related with heterogeneity, despite being in our mind as potential limitations of our model, were left for future work.

(2) The speed of UNSAFE development is a fixed universal constant, and each developer has no control to adjust that speed, but is only able to choose either SAFE (slow) or UNSAFE (fast).

R: We fully agree that it would be interesting to consider that teams can adjust their speed continuously during the race. That would require the introduction of a new dynamical process coupled with this one, which would definitely enlarge the richness of the model. We do, however, believe that the complexity of such coupled system would be beyond the scope of this work. In fact, that idea would be more interesting to address in the absence of any incentives, before combining it with the present framework of rewards and sanctions. We hope the reviewer agrees with us. Nonetheless, we have added a paragraph in the Discussion highlighting this point. Thank you again.

*(3) The competitor developer *can* change the opponent's speed by peer rewarding/punishment (even though it can't change its own speed in a similar way).*

R: Thanks for your comment. Please see our reply above regarding possible examples of peer incentives in an AI development competition.

None of these assumptions seems well justified, especially in the context of AI technology development. Unlike medical and pharmaceutical development that requires extensive clinical testing, technology development in computational domains can happen sometimes at a very rapid pace and result in a breakthrough, not necessarily in a risky technology. The speed of development is most likely not a GO-NOGO binary choice but should be more gradual strategic parameter that each developer can adjust by itself.

R: We agree, but this is not conflicting with our model at all. Indeed, the parameter p_r in our model that captures the level of risk when an unsafe course of actions occurs, can be low which corresponds to the situation where unsafe development (innovation) does not lead to a high-risk technology and is preferred.

Meanwhile, it would be rather difficult to influence the competitor's development speed from the outside, aside from filing lawsuits on, e.g., IP infringements (or perhaps by cyber-attacks).

R: Thank you for your comment. These are also the examples of peer punishment we have in mind (please see the last paragraph in Introduction section). Refuse to share knowledge, or even preventing opponents from accessing common database (see some efforts from EC), would be other ways to influence a competitor's development speed.

Other miscellaneous points:

** Figure 2 was referred to earlier than Figure 1. The order of those figures should be reversed.*

R: Thank you. Done!

** It would be better to re-introduce definitions of symbols (S, B, W, p_r, etc.) in the Materials and Methods section.*

R: Good idea, thank you! Done.

** In page 8: "a cyclic pattern emerges (see Figure 3b)": Figure 3b does not show a cycle.*

R: We consider that a neutral transition has arrows both ways. We have added a clarification on that. Thank you.

** In page 9: "see Figure 5, comparing panels c and f": Figure 5 does not have such panels.*
R: Thank you for point this out. Indeed, Figure *5* should be Figure *3*. We have corrected it. We sincerely apologize for missing this typo.

** In page 9: "This observation confirms the observation": A redundant and unclear expression.*

R: Corrected, many thanks!

** Figure 1 does not have orange or blue areas.*

R: In Figure 1 in the submission (which is now Figure 2 in the revised version), orange and blue areas refer to the two rectangles with the corresponding colour. We've added additional details in the figure caption to clarity these. Thanks.

** The direction of the blue line in Figure 1 does not look correct. Shouldn't its slope be negative, given that the rewarder reduces her speed?*

R: The blue line is bi-directional as a reward indeed decreases the rewarding player's speed but increases the rewarded one's speed. We've added an explanation in the figure caption. Thank you for pointing this out.

We would like to thank the reviewer for her/his time, and for all the suggestions made which certainly helped us to improve our contribution.
